# Fractional Sunburn Threshold UVR Doses Generate Equivalent Vitamin D and DNA Damage in Skin Types I–VI but with Epidermal DNA Damage Gradient Correlated to Skin Darkness

**DOI:** 10.1016/j.jid.2018.04.015

**Published:** 2018-10

**Authors:** Barbara B. Shih, Mark D. Farrar, Marcus S. Cooke, Joanne Osman, Abigail K. Langton, Richard Kift, Ann R. Webb, Jacqueline L. Berry, Rachel E.B. Watson, Andy Vail, Frank R. de Gruijl, Lesley E. Rhodes

**Affiliations:** 1 Centre for Dermatology Research, Division of Musculoskeletal and Dermatological Sciences, School of Biological Sciences, Faculty of Biology, Medicine and Health, The University of Manchester and Salford Royal NHS Foundation Trust, Manchester Academic Health Science Centre, Manchester, UK; 2 Department of Environmental & Occupational Health, Robert Stempel College of Public Health & Social Work, Florida International University, Miami, Florida, USA; 3 School of Earth and Environmental Sciences, The University of Manchester, Manchester, UK; 4 Specialist Assay Laboratory, The University of Manchester, Manchester University NHS Foundation Trust, Manchester Academic Health Science Centre, Manchester, UK; 5 Centre for Biostatistics, Division of Population Health, Health Services Research & Primary Care, School of Health Sciences, Faculty of Biology, Medicine and Health, The University of Manchester, Manchester, UK; 6 Department of Dermatology, Leiden University Medical Centre, Leiden, The Netherlands

**Keywords:** 25(OH)D, 25-hydroxyvitamin D, CI, confidence interval, CPD, cyclobutane pyrimidine dimer, L*, skin lightness, MED, minimal erythema dose, SED, standard erythema dose, SEM, standard error of the mean

## Abstract

Public health guidance recommends limiting sun exposure to sub-sunburn levels, but it is unknown whether these can gain vitamin D (for musculoskeletal health) while avoiding epidermal DNA damage (initiates skin cancer). Well-characterized healthy humans of all skin types (I–VI, lightest to darkest skin) were exposed to a low-dose series of solar simulated UVR of 20%–80% their individual sunburn threshold dose (minimal erythema dose). Significant UVR dose responses were seen for serum 25-hydroxyvitamin D and whole epidermal cyclobutane pyrimidine dimers (CPDs), with as little as 0.2 minimal erythema dose concurrently producing 25-hydroxyvitamin D and CPD. Fractional MEDs generated equivalent levels of whole epidermal CPD and 25-hydroxyvitamin D across all skin types. Crucially, we showed an epidermal gradient of CPD formation strongly correlated with skin darkness (*r* = 0.74, *P* < 0.0001), which reflected melanin content and showed increasing protection across the skin types, ranging from darkest skin, where high CPD levels occurred superficially, with none in the germinative basal layer, to lightest skin, where CPD levels were induced evenly across the epidermal depth. People with darker skin can be encouraged to use sub-sunburn UVR-exposure to enhance their vitamin D. In people with lighter skin, basal cell damage occurs concurrent with vitamin D synthesis at exquisitely low UVR levels, providing an explanation for their high skin cancer incidence; greater caution is required.

## Introduction

Solar UVR induces pivotal effects in skin cells with both negative and positive impact on human health, primarily through genotoxicity, which leads to skin carcinogenesis, and vitamin D synthesis, which is important for musculoskeletal health. Skin cancers are highly prevalent, cause morbidity, and can be fatal (Lomas et al., 2012). The main cause of most skin cancers is accumulation of mutations in genomic DNA after UVR. The UVR damages DNA through photochemical reactions with cyclobutane pyrimidine dimers (CPDs), the prominent mutagenic form of damage. Conversely, UVB (280–315 nm) (Commission Internationale de l’Eclairage, 2011) is essential for photochemical conversion of skin 7-dehydrocholesterol to pre-vitamin D_3_, which is thermally converted to vitamin D_3_; this is usually the body’s main vitamin D source, with few foods naturally containing substantial quantities (Scientific Advisory Committee on Nutrition, 2016). Vitamin D undergoes hepatic hydroxylation to 25-hydroxyvitamin D (25(OH)D), the major circulating form and indicator of vitamin D status, then renal hydroxylation to active hormone 1,25-dihydroxyvitamin D, which promotes calcium absorption in gut and mobilization in bone. Low vitamin D status contributes to rickets and osteomalacia, and epidemiological and experimental studies link vitamin D with prevention of malignant and immune disorders (Ashwell et al., 2010). However, low vitamin D status is reportedly prevalent worldwide (Holick and Chen, 2008).

These issues highlight the desirability of identifying levels of sun exposure that generate 25(OH)D with minimal skin damage, which is further complicated by differences in skin pigmentation. Genetic influence on vitamin D pathway genes, as well as those for melanization, might affect serum 25(OH)D levels, but this is currently unclear (Batai et al., 2014). Melanin effectively absorbs and scatters UVR, influencing the magnitude of its health effects (Clemens et al., 1982, Rijken et al., 2004, Tadokoro et al., 2003). Thus, dark-skinned people are generally less prone to epidermal CPD induction after UVR (Felton et al., 2016, Tadokoro et al., 2003), and there is a higher incidence of melanoma and keratinocyte cancer in light- than dark-skinned people (Gloster and Neal, 2006, Lea et al., 2007). Also, vitamin D deficiency (i.e., 25(OH)D < 25 nmol/L, 10 ng/ml) (Scientific Advisory Committee on Nutrition, 2016) and insufficiency (25(OH)D < 50 nmol/L, 20 ng/ml) (European Food Safety Authority Panel on Dietetic Products, 2016, Institute of Medicine, 2011), as defined by US/Canadian and European authorities, are more common in those of South Asian and African/African Caribbean ethnicity than in white Caucasians (Ford et al., 2006, Kift et al., 2013). Intervention studies show that in contrast to white Caucasians, few South Asians reach sufficiency after repeated low-level simulated sunlight exposures set at an absolute dose of 1.3 standard erythema dose (SED) per exposure (Farrar et al., 2011, Rhodes et al., 2010), although a modest increase in dose can prevent vitamin D deficiency (Farrar et al., 2013).

Sunlight exposure guidance is to remain below levels causing personal sunburn erythema (Cancer Research UK, 2018) while permitting brief exposures to gain vitamin D and is particularly aimed at reducing skin cancer risk in light-skinned people. However, the relationship between vitamin D synthesis and skin cell DNA damage is poorly understood, particularly at UVR doses related to individual sunburn threshold, and across the range of human skin types. Thus, the primary aim of this study was to determine whether 25(OH)D can be gained without, or with minimal, DNA damage induction after acute sub-sunburn UVR doses personalized to sunburn threshold. Further aims were to evaluate whether or not DNA damage persisted after these doses and to examine whether 25(OH)D generation and DNA damage induction and repair are influenced by skin type, given these sunburn threshold individualized doses. To address these, we performed an experimental dose-response study related to personal minimal erythema dose (MED). Well-characterized humans across the range of skin types I–VI (very light to very dark skin) (Fitzpatrick, 1988) were given acute UVR exposures, with direct comparison of relationships for 25(OH)D and CPD production.

## Results

### Characterization of study volunteers

**Figure 1:**
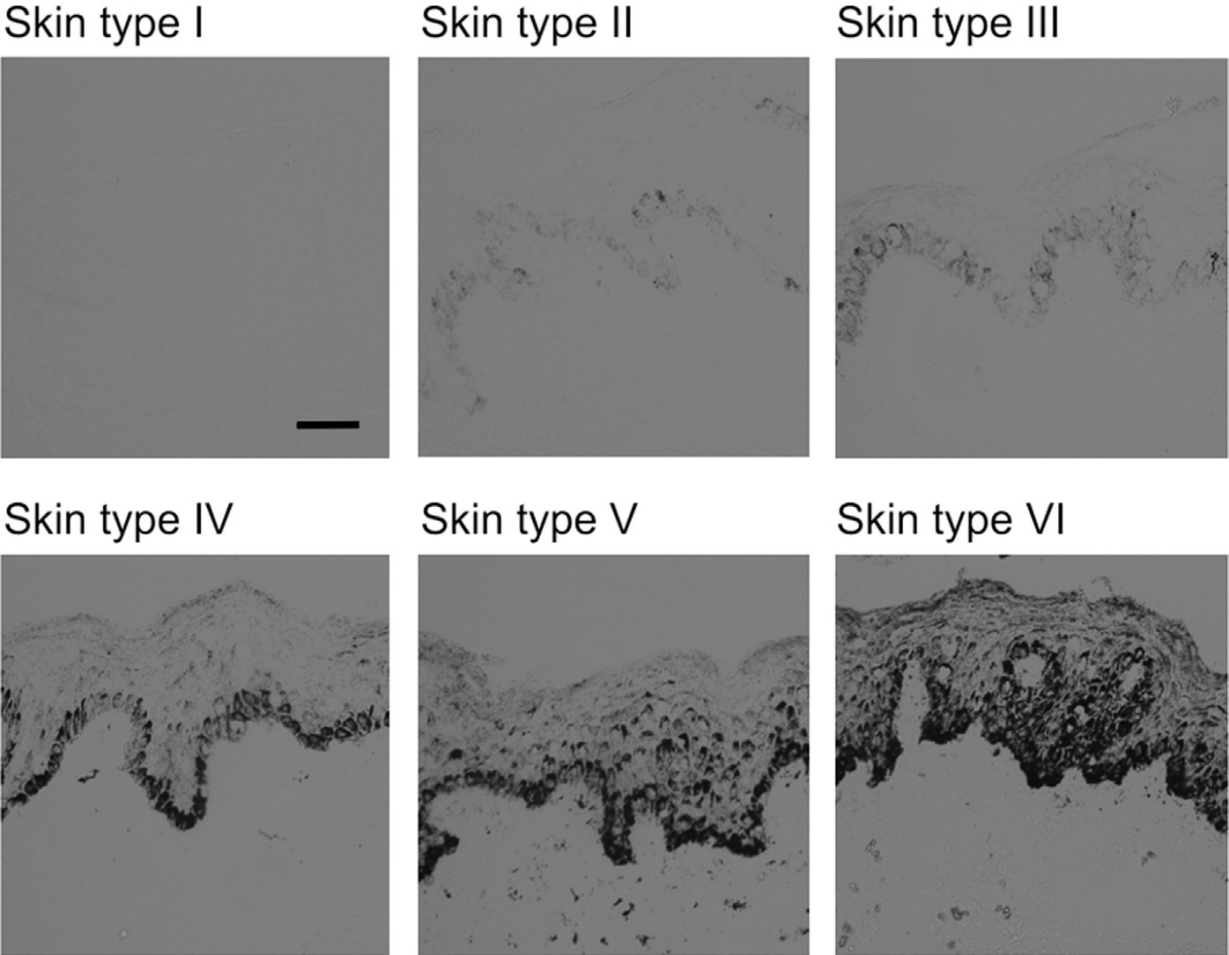
**Constitutive melanin level increases with skin type (I–VI), with differing distribution across the epidermis.** Sections of skin biopsy samples from volunteers with skin types I–VI were stained for melanin using a modified Warthin-Starry procedure. The percentage of epidermis stained for melanin rose with increasing skin type (I–VI). A gradual decrease in melanin staining was seen with increasing distance from the dermal-epidermal junction, although notable amounts of melanin persisted across the full depth of the epidermis in dark skin types. Scale bar = 50 μm.

We performed detailed skin typing of volunteers (n = 39). This involved questions regarding their propensity to sunburn/tan after unprotected sunlight exposure and recording of physical characteristics. Our assessment of skin types corresponded with baseline noninvasive measurements of volunteers’ MEDs and skin lightness (L*; 0 = black, 100 = white) and also with levels of epidermal melanin staining. Thus, a rise in MED was seen, from a mean of 21 (standard error of the mean [SEM] = 1) to a mean of 152 (SEM = 20) mJ/cm^2^ (erythemally effective UVR, equivalent to a mean of 2.1–15.2 SED) across skin types I–VI, accompanied by decrease in L* from 73 (SEM = 1) to 41 (SEM = 2) (Table 1). The wide-ranging L* values showed the inclusion of very light- to very dark-skinned people. Skin biopsy sections showed an increasing percentage of epidermis stained for melanin with skin type (range = 0%–60% in skin types I–VI) (Figure 1) and a strong correlation with noninvasively measured skin darkness, 100–L* (*r* = 0.87, *P* < 0.0001). Overall, melanin levels visibly decreased superficially, that is, with distance from the dermal-epidermal junction. Although there was little/no detectable melanin in the basal and adjacent suprabasal layer and none in upper epidermal layers of the lightest skin types, far higher melanin levels were seen in skin types IV–VI, where melanin clearly persisted across the epidermis (Figure 1). Dietary vitamin D intake was low in all skin types (mean = 2.8 μg/day, SEM = 0.4).

**Table 1:** Volunteer demographics and baseline assessments^1^

Skin Type	n	Age, Years	Sex, F:M	MED,^2^ mJ/cm^2^	L*	Melanin,^3^ %	25(OH)D, nmol/L	Ethnicity/Origin
I	6	35 (5)	1:5	21 (1)	73 (1)	0 (0)	45 (6)	White Caucasian
II	6	25 (2)	5:1	26 (1)	72 (1)	0.28 (0.28)	46 (5)	White Caucasian
III	7	30 (3)	2:5	32 (1)	70 (0)	2.21 (1.44)	50 (8)	White Caucasian
IV	7	30 (3)	5:2	56 (5)	63 (2)	25.14 (4.09)	21 (3)	SE Asian, S Asian, Central/S American
V	7	30 (3)	2:5	75 (7)	50 (3)	42.04 (10.15)	32 (6)	S Asian, African Caribbean
VI	6	33 (4)	5:1	152 (20)	41 (2)	59.70	30 (9)	Black African, African Caribbean
I–III	19	30 (2)	8:11	27 (1)	72 (0)	0.88 (0.55)	47 (4)	As above
IV–VI	20	31 (2)	12:8	91 (11)	52 (2)	34.61 (5.52)	28 (4)	As above
All	39	30 (1)	20:19	60 (8)	62 (2)	12.56 (3.72)	37 (3)	As above

Abbreviations: 25(OH)D, 25-hydroxyvitamin D; F, female, M, male; S, south; SE, southeast.

1 Data are mean (standard error of the mean) unless otherwise stated.

2 Minimal erythema doses are erythemally weighted UVR doses. Doses equate to the following mean standard erythema doses: skin type I, 2.1; II, 2.6; III, 3.2; IV, 5.6; V, 7.5; VI, 15.2.

3 Percentage melanin stained of the whole epidermal area assessed; n = 5, 6, 6, 5, 3, and 1 for skin types I–VI, respectively.

### Gain in 25(OH)D occurs with detectable epidermal DNA damage down to 0.2 MED

**Figure 2:**
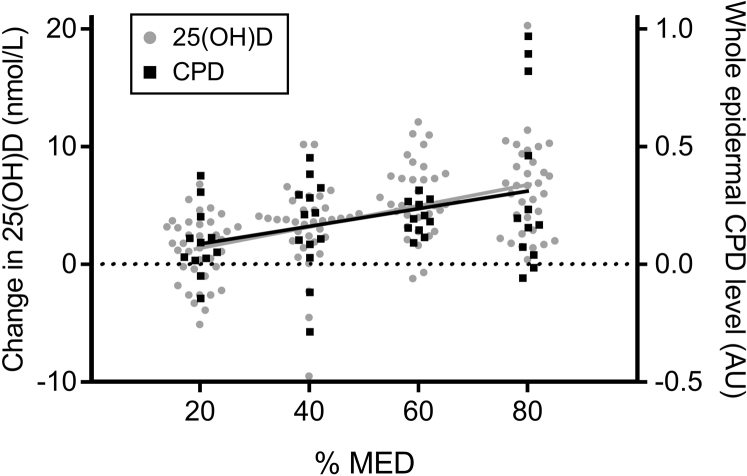
**Sub-sunburn UVR dose responses are seen for 25(OH)D gain and whole epidermal CPD level across skin types I–VI.** Volunteers received an acute UVR exposure of 20%, 40%, 60% and 80% of their individual sunburn threshold dose (MED), and post-UVR serum 25(OH)D change and cutaneous CPD induction outcomes were assessed. A significant response was seen in a mixed-effects regression across the 20%–80% MED dose range for 25(OH)D gain (*P* < 0.001) and CPD level (*P* = 0.01), with no influence of skin type (*P* = 0.23 and 0.63, respectively). There was no evidence of a minimum threshold dose for 25(OH)D gain or CPD induction. Individual data are shown; n = 33–38 per dose for 25(OH)D and n = 11–13 per dose for CPD. 25(OH)D, 25-hydroxyvitamin D; AU, arbitrary unit; CPD, cyclobutane pyrimidine dimer; MED, minimal erythema dose.

To address our primary aim, we performed a dose-response study in which volunteers of all skin types were each exposed to acute sub-sunburn UVR doses of 20%, 40%, 60%, and 80% of their individual MED (see Supplementary Figure S1 online). This equated to the following mean dose ranges of erythemally effective UVR and SED: skin type I, 4.2–16.8 mJ/cm^2^ (0.42–1.68 SED); skin type II, 5.2–20.8 mJ/cm^2^ (0.52–2.08 SED); skin type III, 6.4–25.6 mJ/cm^2^ (0.64–2.56 SED); skin type IV, 11.2–44.8 mJ/cm^2^ (1.12–4.48 SED); skin type V, 15–60 mJ/cm^2^ (1.5–6.0 SED); and skin type VI, 30.4–121.6 mJ/cm^2^ (3.04–12.16 SED). Serum 25(OH)D was assessed before and after each of the four exposures in all volunteers, and epidermal CPD was assessed after either the 20% and 60% or the 40% and 80% exposures. A significant UVR-25(OH)D dose-response relationship was seen with exposures across this dose range (*P* < 0.001; all volunteers) (Figure 2). The observed mean (SEM) gain in 25(OH)D was 1.2 (0.5), 3.3 (0.6), 5.6 (0.5), and 6.4 (0.7) nmol/L after 20%, 40%, 60%, and 80% MED, respectively, with levels returning to baseline after each dose (see Supplementary Table S1 online). Analysis using linear mixed-effects regression showed that pre-UVR 25(OH)D level and UVR dose (as percentage of MED) were significant predictors of post-UVR 25(OH)D level (*P* < 0.001 for both). There was no influence of skin type (*P* = 0.23), with an equivalent change in 25(OH)D seen across skin types. Dark skin-type (IV–VI) volunteers had lower pre-UVR 25(OH)D levels than those with light skin types (I–III), as would be anticipated (Renzaho et al., 2011) (Table 1, and see Supplementary Table S1). The regression estimate of mean UVR effect over the dose range was a 1.6 nmol/L increase in 25(OH)D for every 20% MED increment (95% confidence interval [CI] = 1.1–2.0, all volunteers, adjusted for pre-UVR 25(OH)D level).

We used the same analytical approach to assess epidermal DNA damage data obtained from immunofluorescent staining and quantification of CPD in skin biopsy sections. Because fewer participants contributed biopsy samples, volunteers were grouped into light (types I–III, n = 17) and dark (types IV–VI, n = 9) skin types on the basis of clear differences in skin melanization (Table 1 and Figure 1). A significant UVR-CPD dose-response was seen for the whole epidermis immediately (15 minutes) after exposure (*P* = 0.01, all volunteers) (Figure 2). As with 25(OH)D production, linear mixed-effects regression showed no effect of skin type (*P* = 0.63) on CPD level, with UVR doses personalized to sunburn threshold. The estimated mean UVR effect over the dose range was a 0.13 arbitrary unit increase in CPD for every 20% increase in MED (95% CI = 0.03–0.19, all volunteers). The pattern of response for the 25(OH)D gain and DNA damage were strikingly similar, with no threshold dose seen for either (Figure 2).

### Detectable basal layer CPD occurs concurrent with gain in 25(OH)D in light but not dark skin types after sub-sunburn UVR doses

**Figure 3:**
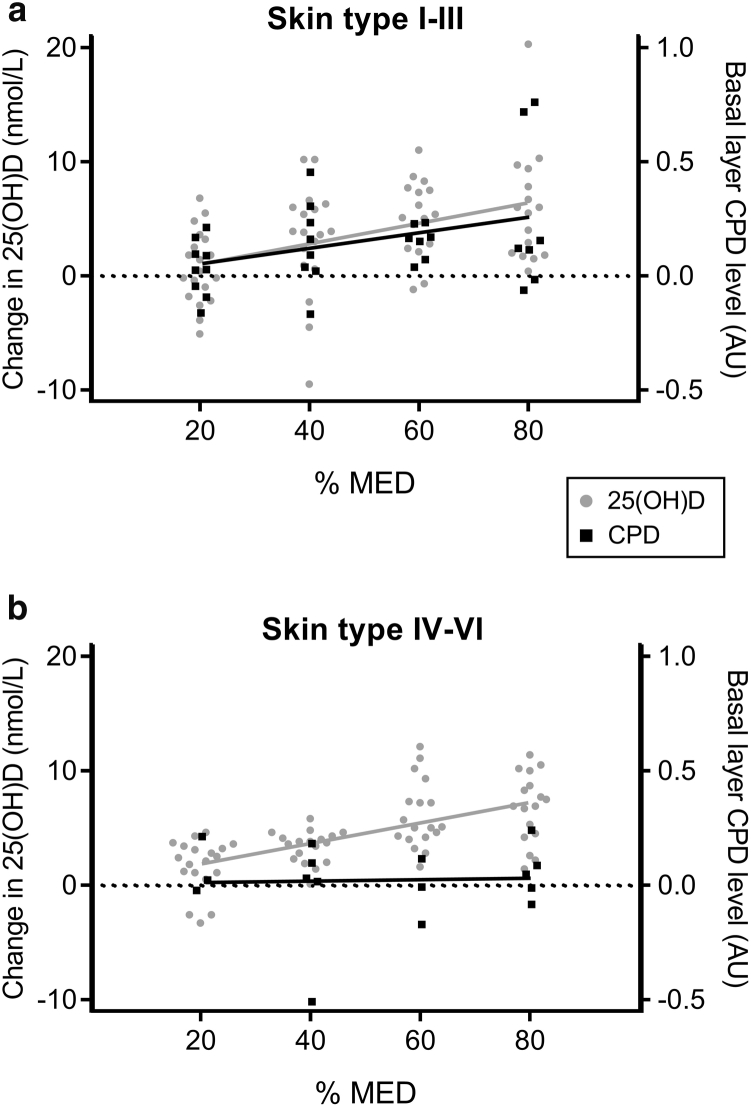
**Gain in basal layer CPD and 25(OH)D occurs concurrently in lighter but not darker skin types.** Assessment of epidermal CPD level in the basal layer alone and serum 25(OH)D was performed in volunteers before and after acute UVR exposures of 20%–80% of their individual sunburn threshold (MED). (**a**) In lighter skin types (I–III), a simultaneous increase in serum 25(OH)D (n = 16–19 per dose) and CPD (n = 7–9 per dose) in the germinative basal layer was seen across the UVR dose range. (**b**) In contrast, in darker skin types (IV–VI), although a similar, significant UVR-25(OH)D dose response occurred, basal layer CPD level remained undetectable across the dose range (25(OH)D n = 17–19 per dose; CPD n = 3–5 volunteers per dose). Individual volunteer data are shown. 25(OH)D, 25-hydroxyvitamin D; AU, arbitrary unit; CPD, cyclobutane pyrimidine dimer; MED, minimal erythema dose.

Having assessed UVR-induced CPD levels across the whole epidermis, we then examined CPD levels in the germinative basal epidermal layer. This layer is the most likely site of skin cancer initiation, containing actively dividing, long-residing cells as opposed to the short-lived, terminally differentiating suprabasal cells moving outward. Regression analysis showed that basal layer CPD level immediately after exposure showed a significant UVR dose-response (*P* = 0.02), with an estimated mean increase of 0.08 arbitrary units for every 20% MED increase (95% CI = 0.01–0.14). In contrast to whole epidermal CPD level, skin type was a significant factor (*P* = 0.03). Thus, both 25(OH)D and basal layer CPD level showed a significant UVR dose-response relationship down to 20% sunburn threshold in light skin types (I–III) (Figure 3a, and see Supplementary Table S2 online), whereas in dark skin types (IV–VI), only 25(OH)D increased with increasing UVR dose, with basal layer CPD being undetectable throughout the 20%–80% MED dose range (Figure 3b, and see Supplementary Table S2).

### CPD distribution shows a gradient across the epidermis that strongly correlates with skin darkness

**Figure 4:**
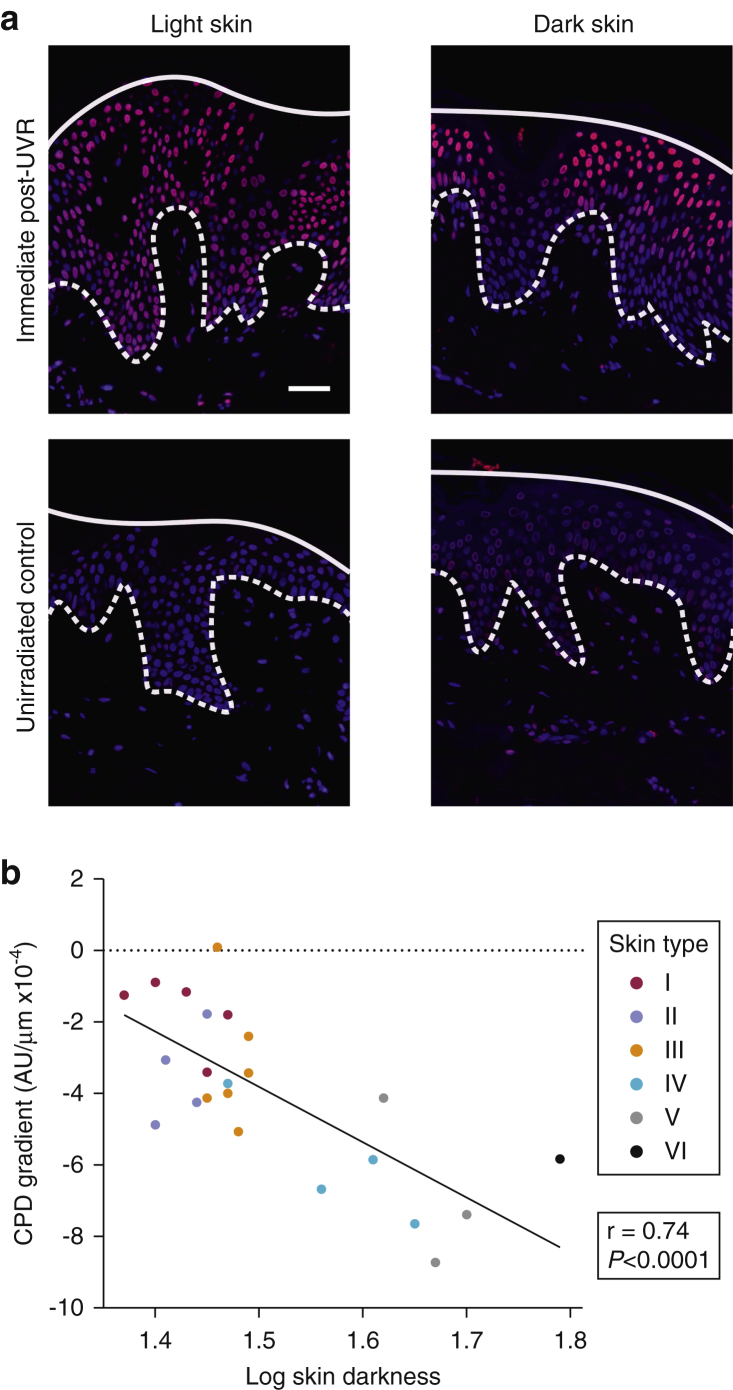
**A gradient of CPD formation is seen across the epidermal depth that strongly correlates with skin darkness.** (**a**) Representative images of CPD (red) and DAPI (blue) staining in skin from volunteers with light (skin type I) and dark (skin type V) skin, immediately after a single 80% MED UVR exposure and in corresponding unexposed control skin. The dashed line indicates the dermal-epidermal junction, and the solid line indicates the skin surface. CPD staining varied little with epidermal depth in light skin, whereas dark skin showed a gradient of CPD formation, with strong staining in the upper epidermis and very little in the basal layer after UVR. Scale bar = 50 μm. (**b**) Total CPD levels were quantified according to epidermal depth by determining the CPD/DAPI ratio within epidermal nuclei along lines perpendicular to the skin surface to generate a CPD gradient value (AU/μm) for each volunteer. The figure shows that the higher skin types had a steeper CPD gradient from skin surface to dermal-epidermal junction than lower skin types and that CPD gradient strongly correlated with skin darkness (100–L*) (*r* = 0.74, *P* < 0.0001). Data points represent volunteers’ gradient values, which are shown for individual skin types. The UVR dose was the highest dose received, that is, 60% or 80% MED. AU, arbitrary units; MED, minimal erythema dose; CPD, cyclobutane pyrimidine dimer.

Because total CPD levels across the whole epidermis did not differ among skin types but basal layer CPD levels were minimal/undetectable across the entire UVR dose range in dark skin, we further explored the influence of skin darkness on the epidermal distribution of DNA damage. For this, we calculated each volunteer’s epidermal CPD gradient, defined as the change (decrease) in CPD level with increasing epidermal depth. In darker skin, the highest CPD levels were found near the skin surface and lowest in the basal layer immediately after UVR, while in lighter skin the CPD distribution was more uniform across the epidermis (Figure 4a). Linear mixed effects regression incorporating all UVR doses showed that this CPD gradient was influenced by skin darkness (100–L*), with steepness of the gradient estimated to increase by 0.0013 arbitrary units/μm for every unit increase in darkness (95% CI = 0.0009–0.0017). The CPD gradient was not influenced by UVR dose (*P* = 0.29). The strong correlation of CPD gradient with skin darkness (*r* = 0.74, *P* < 0.0001) is illustrated for the higher (60% or 80% MED) UVR dose in Figure 4b.

### Epidermal CPD repair is virtually complete and does not differ between skin types after equivalent acute sub-sunburn UVR doses

**Figure 5:**
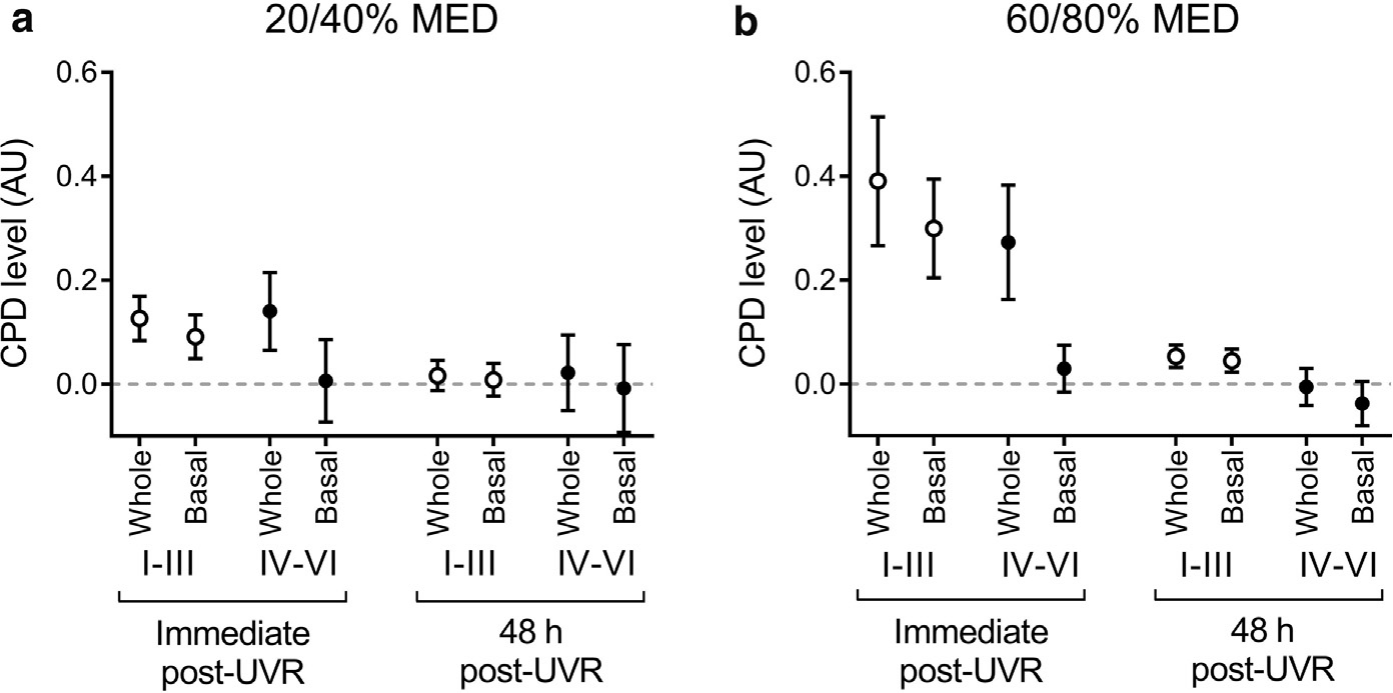
**CPD repair is virtually complete at 48 hours with no difference between UVR doses or skin types.** Epidermal CPD level was assessed immediately (15 minutes) and 48 hours after UVR. Mean whole epidermal CPD level in darker skin types (IV–VI, n = 3–5 per dose) was similar to that in lighter skin types (I–III; n = 7–9 per dose) at both (**a**) the lower (20% or 40% MED) UVR dose and (**b**) the higher (60% and 80% MED) UVR dose with minimal/no CPD detectable 48 hours after UVR exposure. UVR-induced CPD in the basal layer of lighter-skinned volunteers was minimal/undetectable at 48 hours, with CPD in the basal layer of darker skin minimal/undetectable at both time points. Dotted line denotes baseline (unexposed skin); data are mean ± standard error of the mean. AU, arbitrary unit; CPD, cyclobutane pyrimidine dimer; MED, minimal erythema dose.

After examination of DNA damage induction immediately after exposure to sub-sunburn UVR doses, we evaluated whether this damage persisted and if this was influenced by UVR dose or skin type. Assessment of CPD levels at 48 hours after UVR showed that most CPD seen immediately after UVR had been repaired (Figure 5, and see Supplementary Table S2). The level of CPD at 48 hours showed no significant difference from that in unexposed control skin for either the whole epidermis or basal layer and was not influenced by UVR dose (*P* = 0.55 and 0.66, respectively) or skin type (*P* = 0.38 and 0.20, respectively).

## Discussion

The relationship between vitamin D synthesis and DNA damage initiated by sub-sunburn doses of UVR, and how these are influenced by skin type, was previously poorly understood. Through our dose-response study, we have shown that after receiving a range of directly comparable, low sub-sunburn doses of UVR (20%–80% of their personal sunburn threshold dose, MED), volunteers of all skin types gained equivalent serum 25(OH)D and total epidermal CPD levels. Pivotally, however, we quantified the change in CPD level with increasing epidermal depth, that is, the CPD gradient, showing a gradual decrease in CPD level and showing that the steepness of the gradient correlated strongly with skin darkness (100–L*) across the skin types (Figure 4b). Thus, although gaining equivalent 25(OH)D, people with the lightest skin showed little CPD gradient across the epidermis, whereas those with the darkest skin showed a steep gradient, with highest measured CPD levels in the superficial epidermis and virtually no detectable damage in the germinative basal layer, where UVR is most likely to initiate skin cancers. This lack of gradient and higher basal layer levels of CPD in lighter-skinned individuals, despite receiving equivalent sub-sunburn threshold exposures and accordingly far lower absolute UVR doses (with mean doses down to only 4.2 mJ/cm^2^ erythemally effective UVR/0.42 SED in skin type I), provides an explanation for this population’s higher skin cancer incidence.

We found that even the lowest UVR dose (only one fifth of a personal sunburn threshold dose, 0.2 MED) can result in 25(OH)D gain, indicating that very limited exposures can benefit vitamin D status in humans. Our analysis showed that for a single UVR exposure, the mean gain in 25(OH)D was 1.6 nmol/L for each 20% increment in MED, albeit with some interindividual variation (95% CI = 1.1–2.0 nmol/L). The relative ability of dark- and light-skinned individuals to synthesize vitamin D upon UVR exposure has become controversial; although in general, intervention studies suggest that darker-skinned people require higher absolute UVR doses (Armas et al., 2007, Farrar et al., 2013, Xiang et al., 2015), others report a similar response to the same absolute dose (Bogh et al., 2010, Clemens et al., 1982). This discrepancy may relate to protocol differences, including exposures to usually unexposed skin or use of potent medical UVB lamps with higher pre-vitamin D–effective irradiance than sunlight. In contrast to UVB’s role in vitamin D synthesis, the UVA component of sunlight (315–400 nm) (Commission Internationale de l’Eclairage, 2011) promotes reversible conversion of pre-vitamin D_3_ to inactive isomers and even photodegradation of formed vitamin D_3_ within skin (Webb et al., 1989). A further consideration is the possibility that serum 25(OH)D might be controlled at different levels in different populations. Relevant to this concept, black Americans were reported to have lower levels of vitamin D-binding protein in addition to lower 25(OH)D levels than white Americans (Powe et al., 2013). However, it is unclear if this affects bioavailable 25(OH)D; the measurement technique has been questioned (Nielson et al., 2016) and it is not a consistent finding (Yao et al., 2017). We now show that in carefully characterized humans of skin types I–VI, under conditions mimicking natural sun exposure, 25(OH)D production can be predicted and occurs equivalently down to low fractions of individual sunburn threshold doses. This involves higher absolute UVR doses with increasing skin pigmentation (and thus skin type) due to higher MED (Table 1).

Detailed examination of the quantity and distribution of DNA damage (CPD) across the epidermal depth was performed alongside assessment of 25(OH)D gain. We found a dose-response relationship for whole epidermal CPD level at UVR doses of 20%–80% MED. No dose could be identified at which 25(OH)D was produced without detectable DNA damage; CPD were detected even at 20% sunburn threshold, a phenomenon present irrespective of skin type. The UVR dose-response for epidermal CPD is consistent with findings after higher UVR dose series (Fajuyigbe et al., 2018, Young et al., 1996) and shows that this relationship exists even at very low sub-sunburn exposures. Our findings contrast with a report of lower DNA damage in individuals with skin type II than IV given an equivalent 65% MED (Sheehan et al., 2002), although assessing the number of CPD-positive nuclei is less quantitative than our estimation of total CPD levels. Despite comparable total epidermal CPD levels across skin types, the strong correlation we found between skin darkness (100–L*) and epidermal CPD gradient reflected that most damage occurred superficially in dark skin. Although lower levels of DNA damage in the basal layer of dark-skinned volunteers may explain their lower skin cancer risk (Del Bino and Bernerd, 2013, Fajuyigbe et al., 2018, Yamaguchi et al., 2006), further studies are needed to examine the biological consequences of their higher CPD levels occurring in the more superficial layers.

Because both induction and repair of DNA damage influence the risk of mutagenesis, we examined CPD levels immediately (15 minutes) and 48 hours after UVR, a time point appropriate to human studies (Fajuyigbe and Young, 2016). We found that CPD are largely repaired within 48 hours across all skin types; these data were previously lacking for a sub-sunburn UVR dose range in vivo. Although complete repair was seen by this time point, the induced damage could still potentially lead to mutagenesis; future studies could examine the relationship of DNA damage/repair to mutagenesis risk.

To initiate biological effects, sufficient amounts of UVR must reach the relevant epidermal site. Thus, for vitamin D synthesis, UVB photons must be absorbed by 7-dehydrocholesterol for its photochemical conversion. Although 7-dehydrocholesterol is reportedly situated principally within basal and adjacent layers, a smaller amount is in higher epidermis (Holick et al., 1980). Similar amounts of 25(OH)D were produced in people through the skin type range, despite no substantive UVB reaching the basal layer in darker skin, as shown by lack of basal cell CPD. Thus, greater 7-dehydrocholesterol photoconversion appears to have occurred in the upper epidermis, in tandem with evidence of greater UVR effect, as shown by higher CPD levels in upper epidermis of darker skin. Other UVR-induced effects besides mutagenesis may be initiated by CPD, including sunburn erythema (Young et al., 1998). However, although UVR-induced erythema relates to total epidermal CPD level (Fajuyigbe et al., 2018), we show that DNA damage occurs at only 0.2 personal MED, that is, at UVR levels profoundly lower than those causing visibly detectable erythema, highlighting the limitations of using clinical sunburn as a surrogate in sun exposure campaigns.

Melanocytes reside predominately in the basal layer of epidermis, synthesizing melanin, which forms protective caps over keratinocyte nuclei, absorbing and scattering UVR and scavenging UVR-generated radicals (Nielsen et al., 2004, Tadokoro et al., 2003). A recent re-evaluation queried the suitability of melanin assessment by the commonly used Fontana-Masson stain (Joly-Tonetti et al., 2016) and confirmed the enhanced specificity and sensitivity of the modified Warthin-Starry method (Warkel et al., 1980). Combining the latter technique with detailed image analysis, we found that total epidermal melanin levels (as percentage of epidermal area stained) correlated strongly with increasing skin type I–VI, reflecting reported findings for individual typology angle (Hurbain et al., 2018), and that an increasing amount was found in the upper epidermal layers with skin type. Although social/cultural factors (Kift et al., 2013, Renzaho et al., 2011) can influence UVR health effects in different populations, these differences in melanin content across skin types I–VI may be responsible for the steepening CPD gradient we discovered, protecting against risk of skin cancer as skin darkness increases. This is supported by the recent work of Fajuyigbe et al. (2018), who estimated the DNA damage protection factor conveyed by melanin in volunteers at the extreme poles of skin type, that is, skin type VI compared with I/II, after a higher dose series of localized solar-simulated radiation (1.5–12 and 15–120 SED, respectively, for I/II and VI). They found a DNA damage protection factor of 8 for total epidermal CPD in skin type VI versus I/II, and when they assessed the epidermis according to basal layer, middle and upper epidermal regions, the DNA damage protection factor rose to 59 for the basal layer, which was related to the high basal layer melanin content in skin type VI. These observations further suggest that the strong significant correlation of epidermal CPD gradient with skin darkness that we identified through the range of human skin types with low sub-sunburn UVR exposures may also be relevant at high UVR doses and that higher UVR doses may be safely acquired in skin type VI.

Our original work has generated data showing that vitamin D synthesis is initiated at UVR doses as low as one fifth of a personal sunburn threshold dose, but unfortunately, epidermal DNA damage is initiated in tandem. However, our demonstration of a CPD gradient across the epidermis that strongly correlates with skin darkness indicates an increasingly favorable balance of vitamin D and DNA damage responses toward the darker skin types. In contrast, it was seen in lighter skin types that exquisitely low UVR levels produce DNA damage in basal cells, where carcinogenic risk is greatest, offering an explanation for their high skin cancer incidence and challenging guidance on gaining vitamin D “safely” through brief sun exposures below their visible sunburn level. This study informs those formulating public health messages on sun exposure in relation to sunburn, vitamin D acquisition, and skin cancer risk.

## Materials and Methods

### Study protocol and volunteers

The University of Manchester Research Ethics Committee approved the study (reference 11266). Participants gave written informed consent, and the study adhered to Declaration of Helsinki principles. All study procedures were performed at Salford Royal Hospital between November and March (2012–2013 or 2013–2014) when ambient UVB is negligible at northerly latitudes (Manchester, 53.5 °N). The study protocol is summarized (see Supplementary Figure S1) and registered at www.isrctn.org as ISRCTN 97738113.

### Baseline assessments

Detailed characterization of skin type was performed by a modified Fitzpatrick classification (Fitzpatrick, 1988) (see Supplementary Materials and Methods online). This determined history of propensity to sunburn/tan and physical characteristics and placed individuals in categories from very light skin that burns easily and does not tan (skin type I), to black skin (skin type VI). Each volunteer’s skin lightness (L*) (Commission Internationale de l’Eclairage, 2007) score and MED were assessed and confirmed to be consistent with skin type. In dark skin, MEDs were verified using 785-nm laser Doppler speckle contrast imaging (Shih et al., 2014). Epidermal melanin was stained in 7-μm cryosections of unexposed skin using the modified Warthin-Starry procedure (Joly-Tonetti et al., 2016) (see Supplementary Materials and Methods). Melanin level was quantified as percentage of whole epidermis stained (excluding stratum corneum) using Image J software (Abramoff et al., 2004). Oral vitamin D intake was estimated through dietary logs (see Supplementary Materials and Methods).

### Simulated sunlight intervention

A dose series of four UVR exposures related to each individual’s sunburn threshold (i.e., 20%, 40%, 60%, and 80% MED) was given with 1 month between exposures to avoid carryover. Blood was sampled immediately before and 1 week after UVR exposure for 25(OH)D. Three 4-mm buttock skin punch biopsy samples were taken relating to an exposure (unexposed control, 15 minutes and 48 hours after UVR). Because six biopsies were ethically approved per volunteer, a set of three biopsy samples was taken for two exposures (20% and 60% or 40% and 80% MED). Exposures were in a horizontal whole-body cabinet (Philips HB598; Philips, Amsterdam, The Netherlands) fitted with Arimed B (Cosmedico GmbH, Stuttgart, Germany) fluorescent tubes with UVR emission spectrum similar to UK midday June sunlight (95% UVA, 5% UVB); emission was characterized and monitored spectroradiometrically (Rhodes et al., 2010). Approximately 35% of skin surface area was exposed, with volunteers wearing standardized T-shirt and knee-length shorts, mimicking casual summer clothing. A 5 × 10-cm cutout panel in the shorts permitted UVR exposure of an upper buttock area for the purpose of skin biopsy (different buttocks used for each of the two UVR doses, after which biopsy samples were taken). All UVR doses (MED testing and UVR intervention) are given in Commission Internationale de l'Eclairage erythemally weighted mJ/cm^2^, that is, erythemally effective UVR, where 10 mJ/cm^2^ (100 J/m^2^) = 1 standard erythema dose (SED).

### Samples and analysis

All sample analyses were performed at the University of Manchester. Total serum 25(OH)D (D_2_ + D_3_) was determined by liquid chromatography tandem mass spectrometry (see Supplementary Methods and Materials). Immunofluorescent staining for CPD was performed on 3-μm wax-embedded skin sections, with slides blinded for assessment (see Supplementary Materials and Methods). Masks for both whole epidermis and basal cell layer were created, defining the zone of high cellularity in each skin section using CellProfiler (Lamprecht et al., 2007). The dermal-epidermal junction was traced using Matlab (MathWorks, Massachusetts, USA). For each mask, average CPD signal intensity/pixel was measured within each identified nuclear object (DAPI^+^). CPD level was the CPD fluorescence intensity/DNA (DAPI fluorescence) ratio. Six images were taken per sample, three per stained section. CPD/DAPI ratios were log-transformed and averaged to give geometric means; ratio for control skin was subtracted from UVR-exposed samples.

For CPD gradient determination, signal intensity of each pixel (in arbitrary units) was read into Matlab. Second-degree polynomial curves were fitted to the epidermal surface, and perpendicular lines were generated every 10 pixels (image resolution = 0.32 μm/pixel). CPD/DAPI ratio within epidermal nuclei was determined along these lines. K-means clustering created two pixel groups depending on CPD/DAPI ratio. The higher mean cluster was used to determine change in CPD/DAPI ratio using linear regression between log [CPD/DAPI ratio] and skin depth. Images and analyses were examined and quality checks were performed manually; regions with artefacts were excluded.

### Statistics

Primary outcomes were serum 25(OH)D and epidermal CPD levels. Linear mixed-effects regression analyzed post-UVR 25(OH)D in relation to pre-UVR 25(OH)D, UVR dose, and skin type. Linear mixed-effects regression also analyzed CPD level and gradient in skin 15 minutes after UVR in relation to UVR dose and skin type and explored effect of dose by skin type. Analyses were adjusted for repeated measurement by including random effects for volunteers. Because there are no reliable methods for prospective calculation of statistical power for linear mixed-effects models, the rule of thumb of 10 volunteers per parameter estimate was applied. This required a minimum of 30 volunteers to allow simultaneous examination of the effect of dose and skin type on the outcome measures when controlling for baseline. We therefore targeted six volunteers per skin type to ensure a full range and allow for attrition. Statistical analyses used R (www.r-project.org) and Prism 7 (GraphPad Software, La Jolla, CA).

## ORCIDs

Barbara B. Shih: http://orcid.org/0000-0002-3676-3304

Mark D. Farrar: http://orcid.org/0000-0001-8602-7279

Marcus S. Cooke: http://orcid.org/0000-0003-0369-862X

Abigail K. Langton: http://orcid.org/0000-0001-9567-9586

Richard Kift: http://orcid.org/0000-0003-1826-3653

Ann R. Webb: http://orcid.org/0000-0003-2173-0902

Jacqueline L. Berry: http://orcid.org/0000-0003-0107-8275

Rachel E.B. Watson: http://orcid.org/0000-0002-5162-7503

Andy Vail: http://orcid.org/0000-0001-8274-2726

Frank R. de Gruijl: http://orcid.org/0000-0001-6264-3982

Lesley E. Rhodes: http://orcid.org/0000-0002-9107-6654

## Conflict of Interest

The authors state no conflict of interest.
