# Supplementary material for: Fractional Sunburn Threshold UVR Doses Generate Equivalent Vitamin D and DNA Damage in Skin Types I–VI but with Epidermal DNA Damage Gradient Correlated to Skin Darkness
Source: J Invest Dermatol. 2018 Oct;138(10):2244–52. doi: 10.1016/j.jid.2018.04.015 (PMC6158343; doi:10.1016/j.jid.2018.04.015)
Supplement: Supplementary Data [file mmc1.pdf]

SUPPLEMENTARY MATERIAL

Table S1. Serum 25(OH)D level pre- and 1 week post-UVR for doses ranging from 20%-80% MED, in skin type I-VI

Skin type (n/ dose)	25(OH)D (nmol/L)							
	20% MED (n=38)		40% MED (n=36)		60% MED (n=34)		80% MED (n=33)	
	Pre	Post	Pre	Post	Pre	Post	Pre	Post
I (n=6)	44.5(5.9)	44.2(5.4)	37.1(2.5)	38.5(4.9)	33.8(3.8)	38.6(5.2)	35.7(4.7)	41.1(4.1)
II (n=3-6)	45.6(4.6)	48.0(4.6)	37.0(6.0)	43.0(5.8)	31.0(5.4)	38.2(6.0)	33.6(3.7)	39.4(4.2)
III (n=5-7)	49.9(7.9)	50.1(6.8)	42.7(6.8)	45.3(6.4)	38.1(6.3)	42.5(5.9)	34.7(7.4)	41.6(5.9)
IV (n=6)	21.3(3.0)	22.6(2.6)	20.2(2.1)	23.6(1.6)	20.3(1.7)	25.4(1.2)	20.9(1.5)	27.1(1.2)
V (n=5-7)	32.4(6.3)	33.9(6.3)	25.6(4.8)	29.0(4.4)	27.4(4.3)	33.5(4.0)	29.1(5.1)	35.3(4.3)
VI (n=6)	30.3(8.6)	32.8(7.6)	26.9(6.7)	30.2(6.7)	26.0(6.0)	33.1(5.6)	27.4(4.6)	35.2(4.8)

UVR was dosed according to individual’s sunburn threshold (minimal erythema dose, MED).

Data are mean (SEM).

Table S2. CPD level in skin layers for light (I-III) and dark (IV-VI) skin types

UVR dose[1]	CPD level[2]					
	Skin type I-III			Skin type IV-VI		
	Immediate	48 h	% repaired[3]	Immediate	48 h	% repaired[3]
Whole epidermis						
20% MED	0.07(0.04)	0.00(0.04)	-	0.16(0.11)	0.02(0.04)	-
40% MED	0.18(0.07)	0.03(0.04)	64(8)	0.13(0.11)	0.02(0.12)	36(19)
60% MED	0.22(0.02)	0.06(0.03)	64(10)	0.14(0.03)	-0.05(0.06)	90(36)
80% MED	0.50(0.23)	0.05(0.03)	87(5)	0.35(0.17)	0.01(0.04)	90(10)
Basal layer						
20% MED	0.04(0.04)	0.00(0.04)	-	0.07(0.07)	0.00(0.04)	-
40% MED	0.14(0.07)	0.02(0.04)	56(11)	-0.03(0.12)	-0.01(0.14)	-
60% MED	0.16(0.02)	0.05(0.03)	56(17)	-0.02(0.08)	-0.08(0.09)	-
80% MED	0.40(0.18)	0.05(0.03)	82(8)	0.06(0.05)	-0.03(0.04)	-

[1] UVR dosed according to individual's sunburn threshold (minimal erythema dose, MED)

[2] Skin type I-III, n=7-9 for each UVR dose; skin type IV-VI, n=3-5 for each dose. CPD level (arbitrary unit) = CPD/DAPI pixel intensity ratio in UVR-treated - untreated skin. Data are mean (SEM).

[3] % repaired was calculated for each individual at each dose; the value was not calculated where the initial CPD level was ≤ SEM. % repair for samples with the same UVR dose and skin type was not calculated where >30% of the samples showed an initial CPD level ≤ SEM.

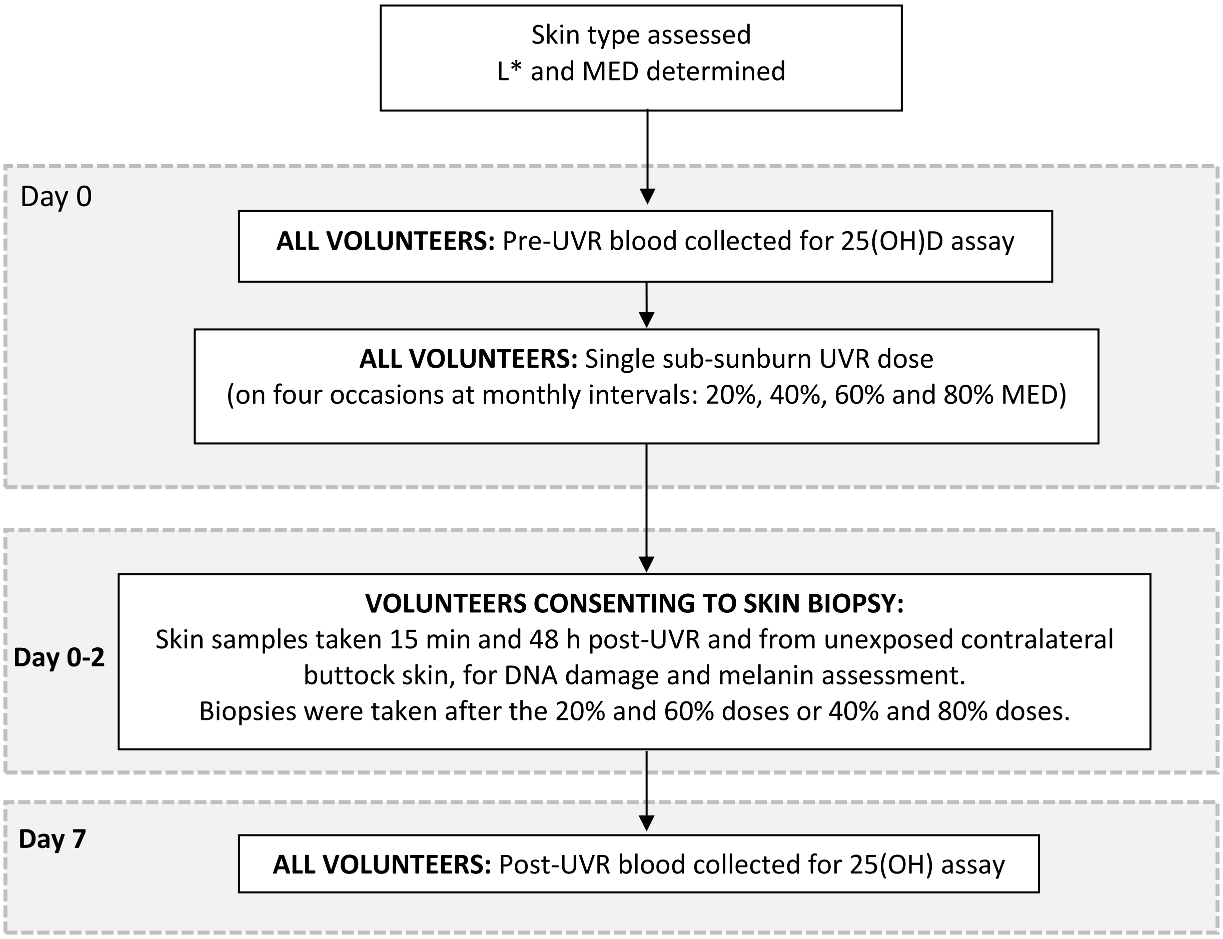

Figure S1. Human study protocol. Following baseline skin assessments, the Day 0 to Day 7 procedures of blood sampling, UVR exposure and further blood sampling were repeated on 4 occasions, once for each UVR dose applied (20%, 40%, 60% and 80% of personal sunburn threshold) with a one month interval between each dose. Skin biopsies were taken on 2 of these occasions i.e. relating to the 20% and 60% doses or the 40% and 80% doses. The study was performed over the winter months to avoid confounding by ambient UVR.

SUPPLEMENTARY MATERIALS AND METHODS

Study protocol and volunteers

Volunteers were recruited by open advertisement. Inclusion criteria: healthy volunteers of skin types I-VI, aged 20-49 y, living in Greater Manchester, UK. Exclusion criteria: history of skin cancer or photosensitivity, sunbathing or sunbed use within 3 months of the study, vitamin D supplementation, photoactive medication, pregnancy and breast feeding.

Baseline assessments

For skin typing, the researcher asked each volunteer standardised questions recalling their responses to UK sunlight: (i) propensity to burn: virtually always, usually, sometimes, rarely/never; (ii) propensity to tan: never/hardly, light tan, mid tan, heavy tan; (iii) response to the first occasion of 30-40 min unprotected exposure to midday June sun; and (iv) evaluated the volunteer for ethnicity; skin, hair and eye colour; presence/absence of freckles. Skin lightness was quantified as L* of the International Commission on Illumination (CIE) L*a*b* colour space on a scale of 0 (black) to 100 (white) (CIE, 2007). Triplicate readings were taken from sun-protected skin (upper buttock, or upper inner arm if lighter in colour) with a spectrophotometer (CM-600D, Konica Minolta). L* was converted to skin darkness (100-L*).

Individual sunburn threshold was assessed as the minimal erythema dose (MED), defined as the lowest dose of UVR producing visually discernible erythema at 24 h post-exposure. A geometric series of 10 erythemally-weighted UVR doses (~30% increments) was applied to sun-protected skin (upper buttock, or upper inner arm if lighter in colour to ensure volunteers did not burn following the subsequent exposures) of each subject (Philips TL-20W/12 broadband UVB lamp). Due to the challenge of erythema perception in dark skin, MED were

verified using near-infrared (785 nm) laser Doppler speckle contrast imaging (Moor Instruments; Shih et al., 2014).

Daily food logs were completed for one week at study commencement to estimate oral vitamin D intake, as described (Farrar et al., 2013). Subjects recorded consumption of foods in seven categories: fortified foods, cheese, butter/spread, milk, red meat, oily fish and eggs. Vitamin D content was obtained from McCance and Widdowson's *The Composition of Food* integrated dataset (Food Standards Agency, 2002), and food package labelling.

Samples and analysis.

Thirty volunteers provided blood samples for all UVR doses, six for three doses and three for one dose. One volunteer was excluded from analysis for non-compliance. Missing samples were due to failure to attend (including four volunteers who withdrew from the study) or insufficient blood drawn. Twenty-seven volunteers provided skin punch biopsies (n=5, 6, 7, 5, 3, 1 for skin types I-VI, respectively) with 23 providing all six, i.e. 3 at both 20% and 60% MED or at 40% and 80% MED, as per the protocol. Three volunteers provided 3 biopsies related to one UVR dose, and one declined further biopsies after the first was taken.

Total serum 25(OH)D (D_2+D_3) was determined by liquid chromatography tandem mass spectrometry (LC-MS/MS) in a Clinical Pathology Accreditation UK (number 0865) laboratory certified proficient by the Vitamin D External Quality Assessment Scheme (Berry et al., 2007; Carter et al., 2004). Intra- and inter-assay CV were 3.7% and 4.8% respectively.

Skin punch biopsies were bisected, half fixed in 10% neutral buffered formalin for 24-72 h before wax embedding, and half snap-frozen in liquid nitrogen. Immunofluorescent staining for CPD was performed on 3 µm wax embedded skin sections. Following dewaxing and rehydration, microwave heat-induced antigen retrieval was performed using citrate buffer (pH 6.0; 10 min, medium-high setting after the solution had boiled). TBS was used for wash

steps. Sections were blocked in 5% goat serum in PBS for 30 min, then incubated overnight with mouse anti-CPD antibody (TDM-2, 1:1500; Cosmo Bio Co., Ltd.) at 4˚C. Incubation with Alexa Fluor 594 goat anti-mouse antibody (1:200) for 40 min at room temperature was followed by counterstaining of nuclei using 4',6'-diamidino-2-phenylindole dihydrochloride (0.2µg/mL, Sigma-Aldrich) for 8 min. Slides were mounted in ProLong Gold Antifade Mountant (Life Technologies), cured for 48-72 h at room temperature then imaged with an Olympus BX51 microscope (20X objective) with Coolsnap ES camera (Photometrics) through MetaVue Software (Molecular Devices). Specific band pass filters (DAPI and Texas red) prevented crosstalk between channels. All images were taken with identical camera settings.

Epidermal melanin staining was performed in 7 µm cryosections of unirradiated skin samples using the modified Warthin-Starry procedure (Warkel et al., 1980; Joly-Tonetti et al., 2016). Cryosections were fixed in acetone for 5 min and rinsed in acidulated water (pH 3.2 achieved using 1% citric acid). Slides were immersed in developer solution (2% silver nitrate, 5% gelatin, 0.15% hydroquinone in acidulated water; Sigma-Aldrich) at 54°C until black colouration was detected (up to 2 min). Slides were rinsed in running hot tap water, then distilled water, and incubated in 5% sodium thiosulphate (in acidulated water) for 2 min. Slides were rinsed in distilled water, dehydrated in a graded alcohol series and permanently mounted. For each skin biopsy, three sections were stained from which three bright field images were captured (Biozero-8000 all-in-one fluorescence microscope; Keyence, Osaka, Japan).

SUPPLEMENTARY REFERENCES

Berry JL, Selby PL, Davies M, Martin J. Observations from the UK Supra-Regional Assay Service laboratory for the measurement of Vitamin D metabolites. J Steroid Biochem Mol Biol 2007;103:477-9.

Carter GD, Carter CR, Gunter E, Jones J, Jones G, Makin HL et al. Measurement of Vitamin D metabolites: an international perspective on methodology and clinical interpretation. J Steroid Biochem Mol Biol 2004;89-90:467-71.

Food Standards Agency. McCance and Widdowson's the composition of foods, 6 edn. Royal Society of Chemistry: Cambridge, UK (2002).
